# Combined optogenetic and electrical stimulation of the sciatic nerve for selective control of sensory fibers

**DOI:** 10.3389/fnins.2023.1190662

**Published:** 2023-06-08

**Authors:** Jerico V. Matarazzo, Elise A. Ajay, Sophie C. Payne, Ella P. Trang, Alex C. Thompson, Jason B. Marroquin, Andrew K. Wise, James B. Fallon, Rachael T. Richardson

**Affiliations:** ^1^Bionics Institute, East Melbourne, VIC, Australia; ^2^Department of Engineering, University of Melbourne, Parkville, VIC, Australia; ^3^Medical Bionics Department, University of Melbourne, Fitzroy, VIC, Australia; ^4^Department of Surgery, University of Melbourne, Fitzroy, VIC, Australia

**Keywords:** sciatic nerve, optogenetics, electrical stimulation, neuromodulation, pain, sensory fiber activation, compound action potential (CAP)

## Abstract

**Introduction:**

Electrical stimulation offers a drug-free alternative for the treatment of many neurological conditions, such as chronic pain. However, it is not easy to selectively activate afferent or efferent fibers of mixed nerves, nor their functional subtypes. Optogenetics overcomes these issues by controlling activity selectively in genetically modified fibers, however the reliability of responses to light are poor compared to electrical stimulation and the high intensities of light required present considerable translational challenges. In this study we employed a combined protocol of optical and electrical stimulation to the sciatic nerve in an optogenetic mouse model to allow for better selectivity, efficiency, and safety to overcome fundamental limitations of electrical-only and optical-only stimulation.

**Methods:**

The sciatic nerve was surgically exposed in anesthetized mice (*n* = 12) expressing the ChR2-H134R opsin *via* the parvalbumin promoter. A custom-made peripheral nerve cuff electrode and a 452 nm laser-coupled optical fiber were used to elicit neural activity utilizing optical-only, electrical-only, or combined stimulation. Activation thresholds for the individual and combined responses were measured.

**Results:**

Optically evoked responses had a conduction velocity of 34.3 m/s, consistent with ChR2-H134R expression in proprioceptive and low-threshold mechanoreceptor (Aα/Aβ) fibers which was also confirmed *via* immunohistochemical methods. Combined stimulation, utilizing a 1 ms near-threshold light pulse followed by an electrical pulse 0.5 ms later, approximately halved the electrical threshold for activation (*p* = 0.006, *n* = 5) and resulted in a 5.5 dB increase in the Aα/Aβ hybrid response amplitude compared to the electrical-only response at equivalent electrical levels (*p* = 0.003, *n* = 6). As a result, there was a 3.25 dB increase in the therapeutic stimulation window between the Aα/Aβ fiber and myogenic thresholds (*p* = 0.008, *n* = 4).

**Discussion:**

The results demonstrate that light can be used to prime the optogenetically modified neural population to reside near threshold, thereby selectively reducing the electrical threshold for neural activation in these fibers. This reduces the amount of light needed for activation for increased safety and reduces potential off-target effects by only stimulating the fibers of interest. Since Aα/Aβ fibers are potential targets for neuromodulation in chronic pain conditions, these findings could be used to develop effective strategies to selectively manipulate pain transmission pathways in the periphery.

## Introduction

Electrical neural prostheses can be used to excite nerve activity for the movement of limbs following paralysis or the treatment of cardiovascular disease, epilepsy, depression, and metabolic disorders, among many other applications (reviewed by Cracchiolo et al., 2021). Activation of peripheral nerve activity *via* application of electrical stimulation to the nerve can also be used to block pain perception in the upper or lower extremities (Van Calenbergh et al., 2009; Deer et al., 2016) or the occipital nerve (Schwedt et al., 2007; Cadalso et al., 2018). One mechanism behind this analgesic effect of electrical stimulation is based on the gate control theory of pain, whereby increased activity of non-nociceptive fibers, such as Aα/Aβ fibers in the sciatic nerve, is known to produce analgesia by inhibiting transmission of pain to second-order neurons through gating at the substantia gelatinosa of Rolando of the dorsal spinal cord (Sheikh and Dua, 2022). Electrical stimulation can be delivered to the nerve *via* transcutaneous electrical nerve stimulation (TENS) or an implanted nerve cuff electrode. However, there is a limited range of current levels that can be applied before unwanted activity is initiated, such as motor fiber excitation, creating a very narrow therapeutic stimulating window. Off-target effects are a fundamental limitation of electrically stimulating most mixed peripheral nerves (Payne et al., 2019a,b), and muscle spasms/cramping are among the many reported adverse events in recipients of nerve cuff electrodes (Dodick et al., 2015; Deer et al., 2016). Lack of selectivity between afferent (sensory) and efferent (motor) fibers, and the functional subtypes of each of these groups of fibers, limits the range of electrical current levels that can be applied before unwanted activity is initiated (i.e., the therapeutic stimulation window). A narrow therapeutic stimulation window reduces efficacy of treatment, and often leads to failure of the technology to translate into the clinic (Byku and Mann, 2016).

Following the emergence of optogenetic techniques in 2005 (Deisseroth, 2011), the potential to modulate neural activity in the peripheral nervous system with genetically introduced light-sensitive ion pumps or ion channels such as channelrhodopsin-2 (ChR2) has emerged as a breakthrough technology to selectively manipulate specific functional types of neurons with light. Using genetic engineering approaches or viral vectors, highly selective expression in subpopulations of a mixed nerve can be achieved *via* cell-specific promoters (Mentis et al., 2006; Llewellyn et al., 2010; Iyer et al., 2014, 2016; Li et al., 2015; Michoud et al., 2021; Fontaine et al., 2021b), or localized injection (Towne et al., 2009, 2013; Booth et al., 2021; Fontaine et al., 2021a). Application of optical stimulation to mixed nerves with selective opsin expression can, therefore, result in selective neuromodulation and avoid the potential off-target effects that may occur during electrical stimulation. Towards clinical realization of optogenetic neuromodulation, clinical trials are underway in the retina to test the safety and tolerability of opsin expression in retinal ganglion cells introduced *via* adeno-associated viruses (GenSight Biologics NCT03326336, Allergan NCT02556736, Nanoscope NCT04945772, NCT04919473).

Despite the therapeutic potential of optogenetics, the power requirements for neural activation *via* optical stimulation in the cochlea are five- to ten-fold higher compared to electrical stimulation (Zierhofer et al., 1995; Hernandez et al., 2014), hindering development of clinically viable devices. In the periphery, large diameter, myelinated axons are more difficult to activate optogenetically compared to small diameter axons due to longer distances between the nodes of Ranvier (Arlow et al., 2013) while the opposite is true for electrical stimulation (Rattay, 1999). Furthermore, moving from rodents to a large animal model (sheep) to better represent the dimensions of human nerves, high intensity light was required for optical activation in optogenetically modified vagus nerve fibers (Booth et al., 2021). The potential for thermal damage with long-term high intensity light has been identified in the retina and the brain (Stujenske et al., 2015; Montazeri et al., 2019; Owen et al., 2019), but has not been well studied in the periphery. Furthermore, neural responses to light are less reliable compared to electrical stimulation (Hart et al., 2020; Thompson et al., 2020; Ajay et al., 2023). Some of these issues may be resolved with rapid development and discovery of new opsins with improved temporal properties sensitivities (Yizhar et al., 2011), but an alternative solution could be achieved by combining optogenetics with electrical stimulation to generate a hybrid response.

During combined stimulation, low intensity light is used to raise the excitability of optogenetically modified neurons, selectively reducing the threshold of electrical activation in these neurons. In isolated ChR2-H134R-modified auditory neurons, a combined stimulus consisting of subthreshold light and subthreshold electrical current as low as 30% of threshold was sufficient to generate action potentials *in vitro* (Hart et al., 2020). These findings were confirmed in the mouse cochlea, whereby combined stimulation with near-threshold light reduced the electrical thresholds for activation more than two-fold, and consequently reduced the spread of electrical activation through the cochlea (Thompson et al., 2020). The greatest influence of combined stimulation on reducing electrical activation thresholds in the hybrid response occurred when the electrical stimulus was delayed relative to the optical stimulus, even when the electrical stimulus occurred after the end of the optical stimulus (Hart et al., 2020; Thompson et al., 2020), in line with the closing kinetics of the ChR2-H134R opsin (Berndt et al., 2011). Compared to optical-only stimulation, combined stimulation also increases the reliability of firing with improved phase locking at higher stimulation rates (Thompson et al., 2020; Ajay et al., 2023).

In this study we examined the effects of combined stimulation in the sciatic nerve, an exemplar mixed somatic peripheral nerve. We used a transgenic mouse with selective expression of the excitatory opsin ChR2-H134R controlled by the parvalbumin promoter. Immunohistochemistry of sensory neurons in L3/L4/L5 dorsal root ganglia (DRG) confirmed expression of ChR2-H134R in sensory afferent Aα/Aβ fibers. Using a nerve cuff electrode with a distal stimulating electrode pair combined with a laser-coupled optical fiber and a proximal recording electrode pair, we measured compound action potentials during optical, electrical, and combined stimulation of the sciatic nerve. Our results suggest that optogenetics can be used to selectively control Aα/Aβ fiber activity in the sciatic nerve. Furthermore, combining optical stimulation with electrical stimulation can increase the recruitment of Aα/Aβ fiber response, increase the therapeutic stimulation window between Aα/Aβ fiber activity and myogenic thresholds, and allow the use of lower optical and electrical stimulation levels. Given the role of Aα/Aβ fibers in pain processing pathways, the results are discussed in relation to chronic pain.

## Materials and methods

### Animals

Experimental mice were derived from the crossing of male COP4*H134R/EYFP mice (Jax strain 012569: B6;129S-Gt(ROSA)26Sor^tm32(CAG-COP4*H134R/EYFP)Hze/J^) and female parvalbumin-Cre mice (PV-Cre; Jax strain 008069: B6;129P2-Pvalb^tm1(cre)Arbr^). All progeny were heterozygous for the ChR2-H134R opsin, expressed *via* the CAG promoter as a fusion protein with enhanced yellow fluorescent protein (EYFP) in cells containing Cre-recombinase *via* the parvalbumin promoter. The use and care of animals in this study follow the Guidelines to Promote the Wellbeing of Animals used for Scientific Purposes (2013), the Australian Code for Care and Use of Animals for Scientific Purposes (8th edition, 2013) and the Prevention of Cruelty to Animals Amendment Act (2015). Mice were kept on a 12-h light/dark cycle and allowed access to standard chow and water *ad libitum*. A total of 12 male transgenic mice were used in this study (preference for male was based on size), at 88–132 days old (average age 107 days). A C57BL/6 wild-type control mouse was also used (77 days old).

### Peripheral nerve cuff electrode

**Figure 1:**
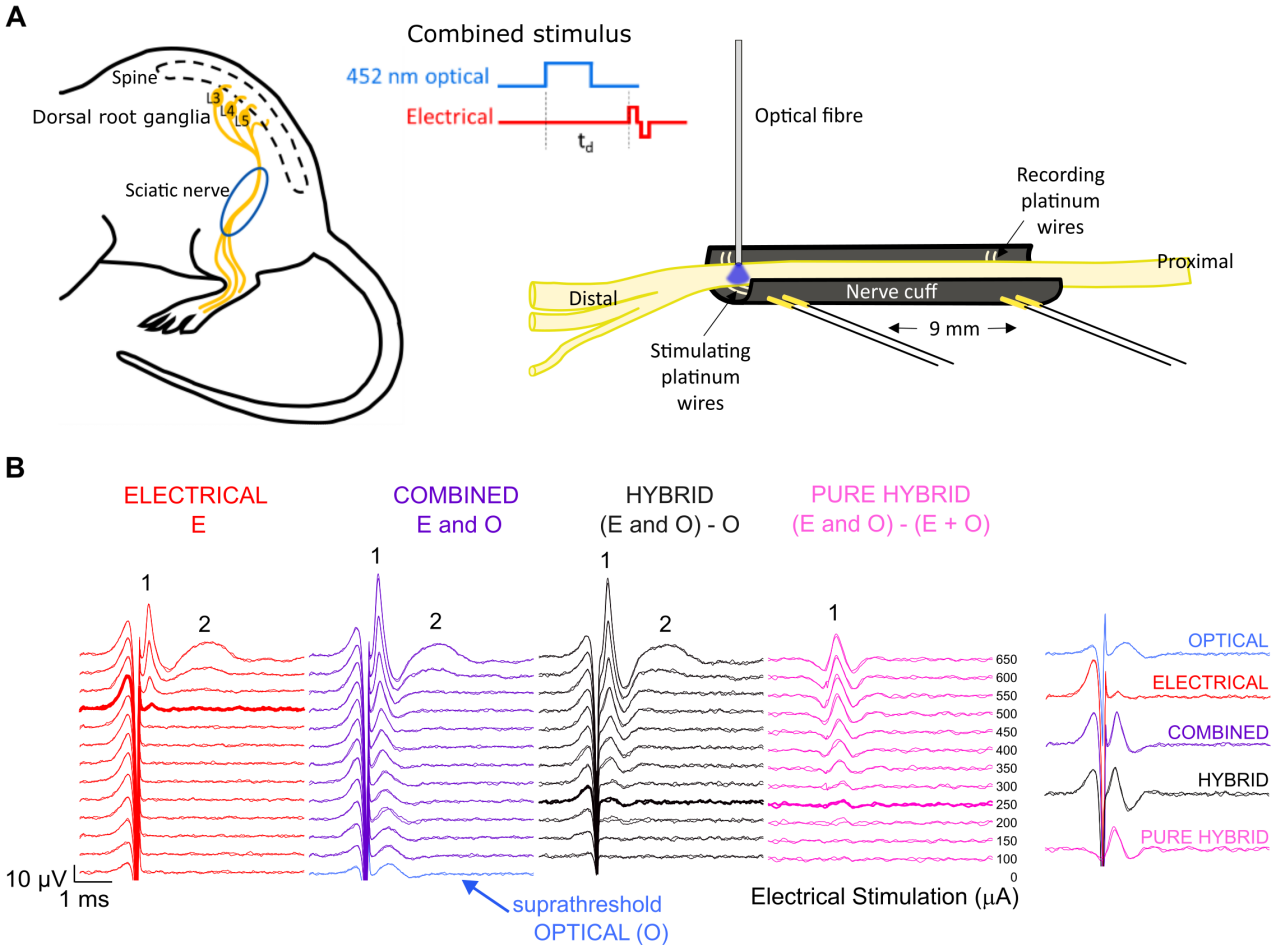
Experimental set-up and analysis. **(A)** The mouse sciatic nerve was surgically exposed between the distal trifurcation and the spine. A channel-shaped nerve cuff electrode containing stimulating and recording electrode pairs spaced 9 mm apart was positioned under the nerve. An optical fiber was positioned <0.5 mm above the stimulating electrode pair to deliver 452 nm laser light. Optical and electrical pulses were delivered individually or as combined stimuli with a variable delay (t_d_) between the onset of the optical pulse and the onset of the electrical biphasic pulse. **(B)** Responses to electrical stimulation (red) and co0mbined stimulation (purple) recorded from the same mouse. For combined stimulation, the stimulus was comprised of optical stimulation (blue, set to a suprathreshold level in this example and visible at 0 μA stimulating current) and a range of electrical stimulation levels delayed 1.5 ms relative to the start of the optical pulse in this example. Subtraction of the optical response from the combined response revealed the ‘hybrid’ response (black), while further subtraction of the matched electrical response at each delivered current level revealed the ‘pure hybrid’ response (pink). The responses to electrical and combined stimulation consisted of a fast-conducting neural response (1) and a slower myogenic response (2). The first visual response above threshold is shown by the bolded traces. A summary of responses is shown at right for 500 μA (the threshold level for electrical-only stimulation).

The channel-shaped nerve cuff electrode was custom-made from premixed MED 4860 and black silica pigment to block the passage of light to underlying muscle. The dimensions of the nerve cuff (outer diameter 3.1 mm, inner diameter 1.8 mm, 12 mm) easily accommodate the mouse sciatic nerve which has a diameter of approximately 0.6–0.8 mm near the trifurcation. The cuff electrode consisted of two bipolar pairs of platinum wire electrodes (100 μm diameter), with electrodes positioned at the bottom of the channel, contacting the full diameter of the ventral side of the overlying sciatic nerve. Adjacently paired electrodes were spaced 0.8 mm apart with 9 mm between the center of each electrode pair (Figure 1A).

### Sciatic nerve cuff electrode implantation and optical fiber placement

Surgical exposure of the left sciatic nerve was performed under gaseous general anesthesia; 1–2% isoflurane (Zoetis, Melbourne, Australia) mixed with oxygen delivered at a flow rate of 1.5 L/min. A heating pad was used to maintain core body temperature, respiration rate monitored over the duration of the experiment (approximately 7 h) and compound sodium lactate solution (Hartmann’s) was injected subcutaneously every 2–3 h. The nerve cuff electrode was implanted around the left sciatic nerve, between the distal trifurcation and the proximal portion of the nerve closest to the spine (Figure 1A). The nerve was infused with warm saline to keep it functionally healthy throughout the recording period and the left leg was secured to minimize movement during myogenic activity. A 105 μm fiber optic with 125 μm silica cladding, 3 mm PVC jacket and numerical aperture of 0.22 (Thorlabs, Newton, NJ) was placed in a 3D-printed electrode holder and positioned 0–0.8 mm from the center of the distal electrode and < 0.5 mm above the sciatic nerve using a manual micromanipulator (Figure 1A). The position of the optical fiber relative to the nerve was visually checked every 10–20 min (the duration of each stimulation run). Myogenic activity was minimized to prevent movement of the optical fiber relative to the nerve.

### Stimulus generators

#### Electrical

A custom-made stimulator was connected to the distal electrode pair of the array for delivery of electrical stimuli (4 Hz, 25 μs pulse width, 8 μs interphase gap; 0–2 mA current; alternating cathodic and anodic polarity). Functionality of all four electrodes was assessed by measuring common ground impedance by passing a biphasic current pulse (100 μA current, 100 μs pulse width) between the active electrode and all others as returns.

#### Optical

The optical fiber was connected to a 452 nm solid state laser (Optotech, Australia) using an FC connector to deliver light pulses (0.25–5 ms duration; 0–23 mW). Irradiance was calibrated with a Fieldmaster power meter and LM10 power meter head (Coherent, Santa Clara, CA). The laser has negligible delay to the trigger input and the time to ramp up to intensity is approximately 25 μs.

### Data acquisition and analysis

#### Acquisition

Compound action potentials (CAPs) evoked by electrical-, optical- or combined stimulation were recorded from the proximal pair of electrodes (averaged over 20 repetitions and 2 repeats presented at 4 Hz) using an isolated differential amplifier with active probe (ISO-80. World Precision Instruments). Recordings were sampled from the proximal electrode pair at a rate of 200 kHz using a data acquisition device (USB-6210, National instruments) and digitally filtered (500–3,000 Hz; IIR Bandpass filter). The neural response threshold to 1 ms pulses of stimulus light was determined visually to set the optical intensity for the combined stimuli to <−5 dB (subthreshold), 0 dB (par), and > 3 dB (suprathreshold) for standardization between animals. In the combined stimuli, electrical stimuli were delivered with a delay of 1–3 ms relative to start of an optical pulse. Electrical-only stimuli were interleaved in a pseudorandom sequence with combined stimulation as controls. In some cases, the sciatic nerve was severed distal to the stimulating electrode pair eliminating myogenic activity evoked by stimulation of efferent motor nerve fibers.

#### Post-recording analysis

To reduce the impact of electrical artifact on the visibility of the ECAP response, post-analysis traces were reviewed using backward filtering and traces with alternating stimulation polarity averaged. Example traces are shown in Figure 1B. To compare combined stimulation to electrical-only stimulation, the optical-only response (blue; suprathreshold in this example) was subtracted from the combined response (purple) to obtain a ‘hybrid’ response (black; Figure 1B). Subtracting both the electrical-only and optical-only components of the combined response enables visualization of the ‘pure hybrid’ response, i.e., the facilitation of the optical and electrical stimuli (Figure 1B). Hybrid responses (i.e., responses to combined stimulation with the optical component subtracted) were used to directly compare to electrical-only stimulation for all analyses. The neural response was measured using peak-to-peak analysis and threshold was defined as the minimum stimulus intensity producing a response amplitude of at least 0.1 μV above background (Fallon and Payne, 2020) within a post-stimulus latency window of 0.3–0.6 ms.

### Histology

Mice were deeply anesthetized with 350 mg/kg pentobarbital sodium (i.p.) prior to intracardial perfusion with warmed 0.9% (w/v) saline followed by 10% (v/v) neutral buffered formalin at 4°C. The left sciatic nerve and left and right L3/L4/L5 dorsal root ganglia (DRG) were removed, post-fixed for 24 h at 4°C and then washed in phosphate buffered saline (PBS). Tissues were cryoprotected in 30% sucrose before being embedded in optimal cutting temperature compound (OCT, Tissue-Tek, Sakura, Japan) and sectioned at 20 μm using the CryoStar NX70 Cryostat (Erpedia, MI, United States) at −20°C. Sections were washed twice in PBS and blocked for 3 h at room temperature with PBS containing 2% (v/v) donkey serum and 0.3% (v/v) Triton X-100 prior to overnight incubation (4°C) in chicken anti-neurofilament 200 (NF-200, 1:200, AB5539, Merck Millipore, Australia) antibody and goat anti-calcitonin gene related peptide (CGRP, 1:200, 1720–9,007, BioRad, CA, United States) antibody diluted in PBS containing 2% (v/v) donkey serum and 0.3% (v/v) Triton X-100. Sections were washed in PBS three times prior to 3 h room temperature incubation in the following fluorescent secondary antibodies: Alexa Fluor donkey anti-chicken 647 (1:500, A78952, Thermo Fisher Scientific); and Alexa Fluor donkey anti-goat 594 (1:500, A11058, Thermo Fisher Scientific), both diluted in the solution as per the primary antibodies. Sections were washed three times in PBS prior to coverslipping with Vectorshield anti-fade mounting media (Vector Laboratories, CA, United States) and imaged using a Zeiss Axioplan II microscope (Carl Zeiss Microscopy, Jena, Germany) and AxioVision Software (Zeiss, New York, United States). Selected slides were also imaged on a confocal microscope (Nikon A1R) using 20x and 60x magnification lenses. A maximum projection of 20–30 Z-plane slices, imaged at 1 μm apart, was applied to the images.

Images were loaded into ImageJ software (Rasband, W.S., ImageJ, U. S. National Institutes of Health, Bethesda, Maryland, United States, https://imagej.nih.gov/ij/, 1997–2018) for cell counts. Thresholding was used to differentiate features of interest from background, using the default threshold setting and adjusting the minimum threshold value to 10–15%. DRG cell counts and area measurements were made at L3, L4 and L5 from the left side of 2–3 mice (8 DRGs in total: 2x L3, 3x L4, 3x L5). Total cells, YFP-positive cells, CGRP-positive cells and NF200-positive cells were counted to determine the percentage of YFP-positive cells in the DRG, and the proportion of YFP-positive cells that co-labelled with CGRP and NF200 (averages taken from 2–3 sections per DRG). Area measurements of YFP-positive, NF200-positive cells, and cells that co-labelled with YFP and NF200 were taken (4–17 cells per section).

### Statistics

All data was normally distributed. A one-way repeated measures (RM) analysis of variance (ANOVA) was used to assess the effect of delay on the P1 threshold of combined stimulation compared to electrical only and therapeutic stimulation window increase. Additionally, the effects of optical intensity on relative P1 threshold and response size increase were tested using one-way RM ANOVAs. Tukey’s *post-hoc* comparisons procedure was used for multiple comparisons in one-way RM ANOVAs. Following one-way RM ANOVAs, data was statistically tested against no change using paired t-tests with Bonferroni correction for multiple-comparisons. In some data sets, subjects that did not have data across all delays were removed to allow for a repeated measures analysis. Significance was accepted if *p* < 0.05, with adjustments made to the threshold *p* value for multiple comparisons (adjusted value after correction reported alongside the *p* value). Analysis of ChR2-H134R expression and soma diameter measurements from immunohistochemical sections are presented as mean ± standard error of the mean.

## Results

### ChR2-H134R expression in the sciatic nerve and DRG neurons

**Figure 2:**
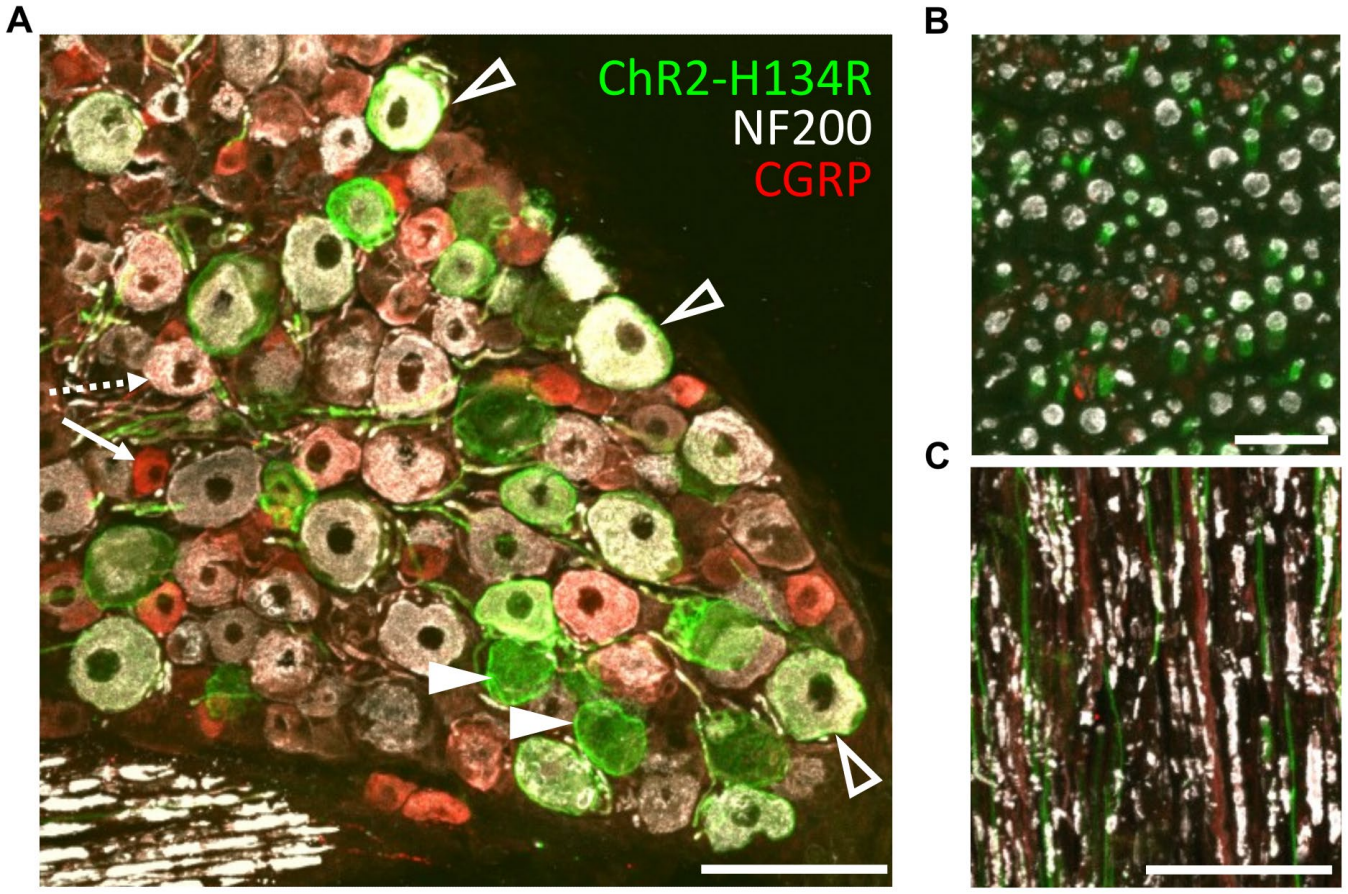
Phenotyping of ChR2-H134R/EYFP expressing neurons in L3/L4/L5 DRGs. **(A)** Confocal image of a cross section through a DRG from the ChR2-H134R mouse showing ChR2-H134R/EYFP expression (green) in a subset of NF200-positive neurons (white, open arrowheads) and NF200-negative neurons (closed arrowheads). ChR2-H134R was not detected any CGRP-positive neurons (red) such as the small-diameter peptidergic nociceptive neurons (arrow) that are likely to give rise to unmyelinated C fibers, and the larger diameter CGRP-positive neurons that co-labelled with NF200 (dashed arrow) that are likely to give rise to Aδ fibers. Scale bar is 100 μm. **(B)** Cross section and **(C)** longitudinal section of the sciatic nerve, showing CHR2-H134R (green) in a subset of NF200-positive fibers (white). The tissue was also stained with CGRP antibodies (red). Scale bars in **(B,C)** are 20 μm and 100 μm, respectively.

The transgenic mice (PV-Cre x ChR2-H134R/EYFP; hereon referred to as ChR2-H134R mice) were characterized by immunohistochemical examination of L3/L4/L5 dorsal root ganglia (DRG) and the sciatic nerve. ChR2-H134R expression was visualized *via* the fused enhanced yellow fluorescent protein (EYFP) tag. An antibody to NF200 was used to detect medium-large diameter myelinated DRG neurons of which a subpopulation is associated with pain transmission, and a CGRP antibody was used to identify peptidergic nociceptors (Ishikawa et al., 2005). ChR2-H134R was expressed in 28.5 ± 3.1% of the total DRG cells at L3/L4/L5 (n = 3; Figure 2A). Within the ChR2-H134R-positive population, 44.5 ± 3.2% of YFP-positive DRG neurons expressed NF200 and had a neuronal area of 1,011 ± 118 μm^2^. Those that did not express NF200 were 688 ± 108 μm^2^ in area (Figure 2A). Less than 1% of YFP-positive cells co-expressed CGRP. Cross sections and longitudinal sections through the sciatic nerve also demonstrated ChR2-H134R expression in a subset of the NF200-positive fibers (Figures 2B,C).

### Electrical stimulation of the sciatic nerve

**Figure 3:**
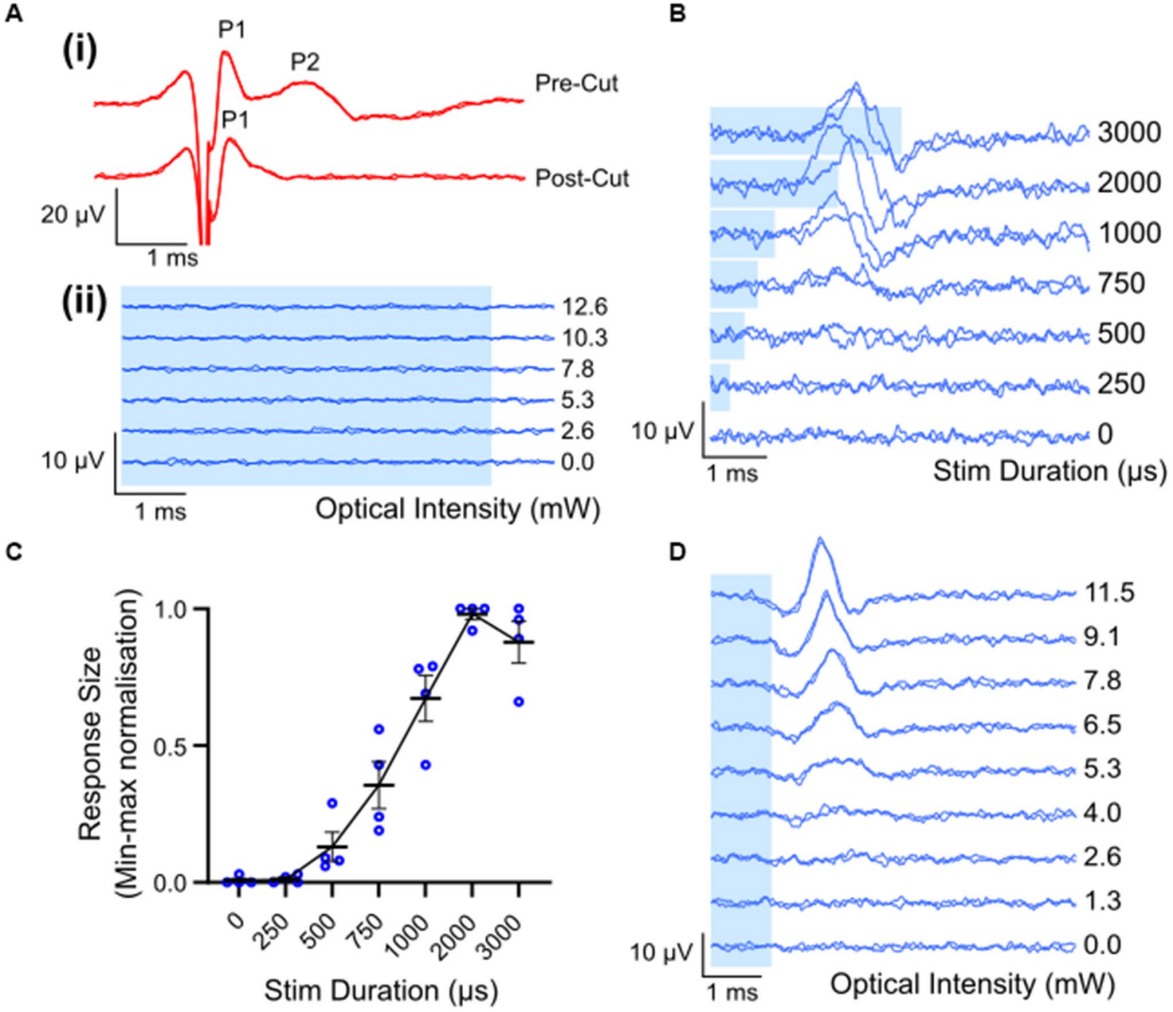
Electrically and optically evoked compound action potentials (ECAPs/OCAPs) in the sciatic nerve. **(A)** Control experiments. **(i)** ECAPS recorded from an intact sciatic nerve during stimulation with 400 μA current in ChR2-H134R transgenic mice, as well as from the same nerve after severing the distal portion of the nerve. **(ii)** No response to optical stimulation was observed in a wild-type C57BL/6 mouse at any pulse duration or any optical power (5 ms optical pulse shown in this example, up to 12.6 mW). **(B)** OCAP responses to 6.8 mW optical stimuli ranging in pulse width from 250–3,000 μs. A trace for no light is also shown. **(C)** Input–output curve for optical stimulus duration and normalized peak-to-peak response size (n = 4; error bars represent standard error of the mean). **(D)** Typical OCAPs in response to 1 ms optical pulses, as recorded from one ChR2-H134R-eYFP transgenic mouse. Blue shading in **(A,B,D)** represent the optical stimulation time.

Waveforms evoked by electrical stimulation exhibited two distinct peaks: a fast-conducting, low-threshold neural peak (P1) that could be separated from the stimulation artifact, and a motor response (P2) with a longer latency picked up from gross muscle activity in the leg that was not present after severing the nerve distal to the site of stimulation (Figure 3Ai). The average threshold (defined as the minimum stimulus intensity to evoke a response amplitude of at least 0.1 μV above background) for P1 was 493 ± 46 μA (range 258–700 μA, *n* = 8). The latency of the P1 waveform was centered at 0.33 ± 0.01 ms, making the average conduction velocity 28.1 ± 1.6 m/s (range 24.6 to 38.6 m/s; *n* = 8). There was a minimal intra-animal drift of ECAP thresholds of 50 ± 13.4 μA (range 0–100 μA; *n* = 8) during the 5 to7 h recording period. Myogenic activity, i.e., P2, was detected at only 70 ± 29.1 μA above the electrical threshold (range 0–142 μA, *n* = 5). Myogenic thresholds were consistent during experiments, deviating on average 20 ± 12.2 μA (range 0–50 μA; *n* = 5). Additional, high threshold neural responses with a conduction velocity of 1.3 m/s (*n* = 1) were detected when the recording electrodes were positioned closer (6 mm) to the stimulating electrodes, using a longer duration electrical pulse and higher stimulating currents (data not shown).

### Optical stimulation of the sciatic nerve

Optical stimulation applied to the sciatic nerve of a wildtype mouse (*n* = 1) did not elicit detectable optically evoked compound action potentials (OCAPs) at any of the optical power levels or pulse durations used (Figure 3Aii). In ChR2-H134R transgenic mice, OCAPs with a single peak could be elicited in the sciatic nerve in response to 500–3,000 μs duration optical stimuli, but not 250 μs stimuli, for powers above 9.6 ± 1.9 mW (*n* = 4). An example from one mouse is shown in Figure 3B for 6.8 mW optical stimuli. The response size increased with increasing stimulus duration and reached a maximum for 2000 μs (*n* = 4) (Figure 3C). Similarly, OCAP response size also increased with increasing optical intensity, with an example from one mouse shown in Figure 3D. No myogenic activity was recorded in any animals during optical stimulation (*n* = 12). High intensities (e.g., 15 mW) or long pulse durations (e.g., 5 ms) often resulted in loss of the OCAP for that part of the nerve. Based on this, 1 ms light pulses below 15 mW were used for all further optical and combined stimulation protocols.

The average latency of fiber populations responsive to a 1 ms light pulse was 1.97 ± 0.06 ms (range: 1.7–2.2 ms; *n* = 8). Due to the relatively long optical light pulse used, it was difficult to accurately determine when the OCAP starts, hence conduction velocity was calculated by positioning the optical fiber 2 mm distal to the stimulating electrode pair and using the two electrode pairs (refer to Figure 1A for position of electrode pairs) as recording sites. The peak response time difference across the two recording sites was compared and conduction velocity was calculated to be 34.3 ± 8.6 m/s (*n* = 2).

### Combined stimulation of the sciatic nerve

#### Effect on P1 threshold

**Figure 4:**
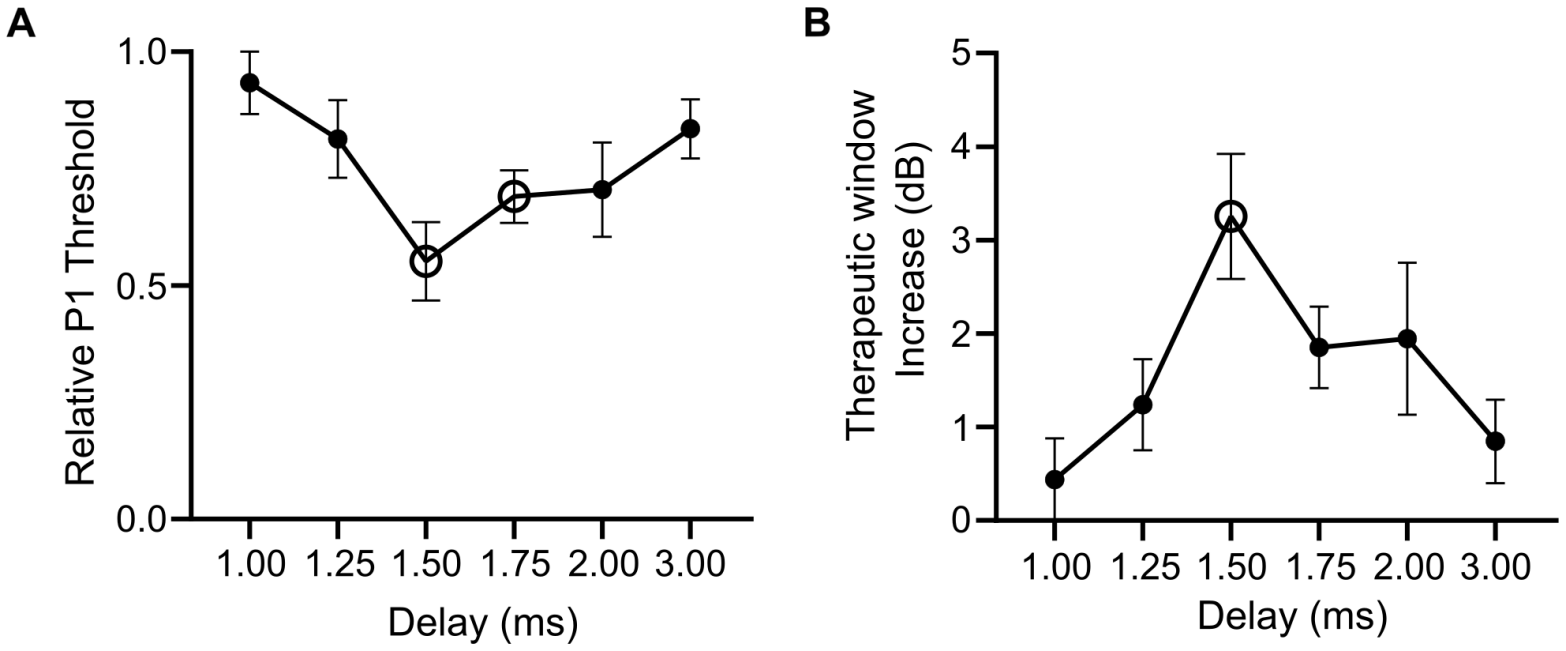
Relative changes in electrical threshold and therapeutic stimulation window during electrical-only and combined stimulation in mice expressing ChR2-H134R. **(A)** Threshold stimulating current for P1 elicited by combined stimulation (with suprathreshold optical stimuli) relative to electrical-only stimulation. During combined stimulation, the electrical stimuli were presented at varying delays (1.00–3.00 ms). Open symbols indicate a significant reduction in relative P1 threshold compared to no change in P1 threshold (1.50 ms *p* = 0.006, 1.75 ms *p* = 0.005, paired t-test, threshold *p* value after Bonferroni correction *p* < 0.008, *n* = 5). **(B)** Therapeutic stimulation window increase between electrical-only stimulation and combined stimulation at varying delays. Open symbol indicates a significant increase in therapeutic stimulation window (1.50 ms *p* = 0.008, paired t-test, threshold *p* value after Bonferroni correction *p* < 0.008, n = 4). Data show mean ± SEM.

To examine how combining optical and electrical stimuli influences the response of the sciatic nerve, we compared the minimum electrical current required to elicit the P1 response (P1 threshold) in suprathreshold combined stimulation (in which the optical stimulus was set to >2.5 dB of optical only threshold) relative to electrical-only stimulation. The electrical stimulus was also delayed 1–3 ms from the onset of a 1 ms optical pulse in combined stimulation to evaluate the effect of delay when facilitating a hybrid response to combined stimulation. A reduction in the P1 threshold was apparent in the hybrid (and pure hybrid) responses to combined stimulation relative to electrical-only stimulation. The largest change occurred when electrical stimulation was delayed 1.50 ms during combined stimulation resulting in a 46.7% reduction of the minimum electrical current required to elicit the P1 response (Figure 4A). There was a trend towards an effect of delay on P1 threshold, but this was not significant (*p* = 0.08, one-way RM ANOVA, *n* = 5). To determine if the change in threshold that occurred during combined stimulation was significantly different to no change in threshold, we compared relative P1 threshold at each delay to a relative change of 1.0 (i.e., no change). It was found that the 1.50 ms delay and the 1.75 ms delay had significant reductions (*p* = 0.006 and *p* = 0.005 respectively, paired t-test, adjusted statistical power after Bonferroni correction for multiple comparisons was *p* < 0.008).

#### Effect on therapeutic window

Next, we evaluated the influence of combined stimulation on the fast-conducting P1 threshold reduction in animals where P2 myogenic activity was maintained (n = 4) and a therapeutic stimulation window could be measured (the stimulating current difference between P1 threshold and P2 threshold in dB). Electrical-only stimulation and combined stimulation were used with variations of the delay in the electrical stimulus delivered during combined stimulation (1.00–3.00 ms). During combined stimulation, we observed an increase in the therapeutic window between the thresholds for the P1 and P2 responses. The largest shift was observed at 1.50 ms electrical delay during combined stimulation where a 3.25 ± 0.67 dB increase in the therapeutic stimulation window was observed compared to electrical-only stimulation (Figure 4B). No effect of delay on therapeutic window was observed (*p* = 0.12, one-way RM ANOVA, n = 4). Analysis of the increase in therapeutic window occurring due to combined stimulation was tested against no increase at each electrical delay, where 1.50 ms was found to result in a significant increase in therapeutic window (*p* = 0.008, paired t-test, adjusted statistical power after Bonferroni correction for multiple comparisons was *p* < 0.008).

#### Effect of optical intensity

**Figure 5:**
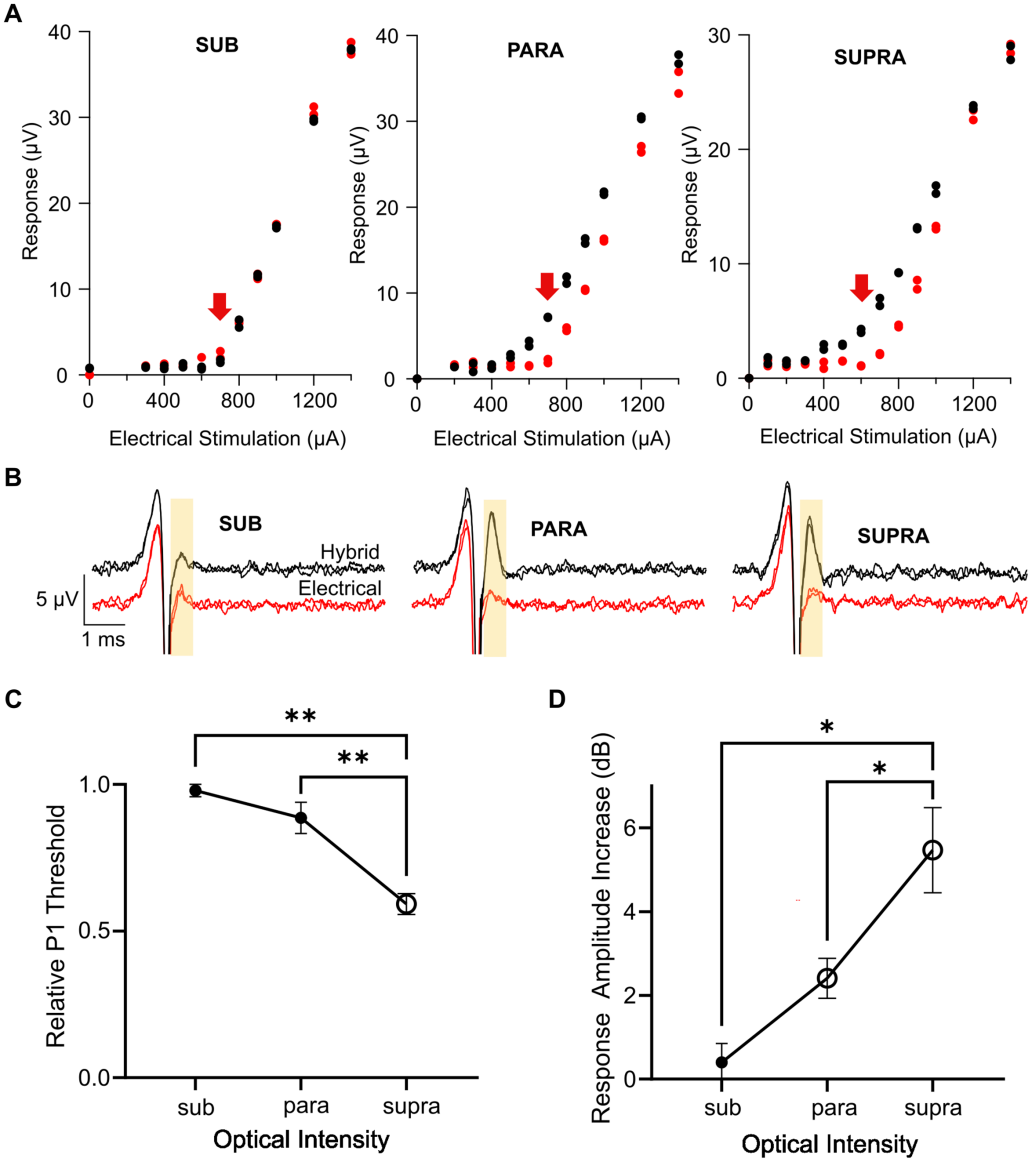
Effect of optical intensity in the combined stimulus on the hybrid response. **(A)** Example response curves for the P1 ECAP in one mouse during electrical-only stimulation (red) or combined stimulation (black) in which the optical intensity was set to subthreshold, parathreshold, or suprathreshold intensities and the electrical stimulus delayed 1.5 ms relative to the optical stimulus. Red arrow indicates electrical threshold. **(B)** Example P1 ECAP trace at the electrical threshold stimulating current (arrow in **A**) for electrical-only stimulation (red) and combined stimulation (black) in which the optical intensity was set to subthreshold, parathreshold, or suprathreshold intensities. The analysis window is shown by the yellow shaded area. **(C)** P1 threshold for combined stimulation relative to electrical-only stimulation. The optical intensity during combined stimulation was set to subthreshold, parathreshold, or suprathreshold levels. The reduction in relative P1 threshold was significantly greater using suprathreshold optical intensities compared to parathreshold or subthreshold optical intensities (^**^p < 0.01, one-way RM ANOVA, *n* = 6). Open symbol indicates a significant decrease in relative P1 threshold compared to no change in threshold (supra *p* < 0.001, paired t-test, threshold *p* value after Bonferroni correction *p* < 0.016, *n* = 6). **(D)** P1 amplitude increase between electrical-only stimulation and combined stimulation at the three optical intensities. The increase in response amplitude was significantly greater using suprathreshold optical intensity in the combined stimulus compared to parathreshold or subthreshold optical intensities (^*^p < 0.05, one way RM ANOVA, *n* = 6). Open symbols indicate significant increases in response amplitude compared to no change in response amplitude for parathreshold optical intensity and suprathreshold optical intensity (*p* = 0.004 and *p* = 0.003 respectively, paired *t*-test, threshold *p* value after Bonferroni correction *p* < 0.016, *n* = 6). Data in **(C,D)** show mean ± SEM and individual data points.

Key advantages of combined stimulation are that the light can be used at near-threshold levels and the electrical current can be delivered at lower current levels, thus keeping the optical power levels low, reducing the reliance on the optogenetic ion channels, and reducing the risk of side-effects from the electrical stimulation. We examined three different combined stimulation protocols, where the intensity of light used in the combined stimulus was set to subthreshold (<−5 dB), parathreshold (0 dB), or suprathreshold (>2 dB) intensities while the electrical stimulus was delivered at a range of intensities below and up to myogenic threshold. The delay between the onset of the optical and electrical stimuli was set at 1.5 ms. Compared to electrical-only stimulation, the response curves (P1 response size against electrical stimulation current) for combined stimulation were shifted to the left when using parathreshold and suprathreshold light, but not subthreshold light, indicating a reduction in threshold for these combined stimulation intensity levels (Figure 5A). The main impact on nerve fiber recruitment, as shown by the P1 peak-to-peak response amplitude, occurred near electrical threshold (Figure 5B).

Comparing the P1 threshold during combined stimulation relative to electrical stimulation for the three optical intensities, there was a significant effect of the optical intensity used during combined stimulation (*p* < 0.001, one-way RM ANOVA, *n* = 6; Figure 5C). *Post-hoc* analysis showed the relative P1 threshold for suprathreshold optical intensity was significantly different to the relative P1 threshold using parathreshold (*p* = 0.004) and subthreshold (*p* = 0.002) optical intensities in the combined stimuli. Relative P1 threshold for each of the combined stimulation optical intensities was then tested to no change (i.e., a relative P1 threshold of 1.0) where it was found that combined stimulation with suprathreshold optical intensity yielded a statistically significant reduction in threshold relative to electrical stimulation only (*p* < 0.001, paired t-test, adjusted statistical power after Bonferroni correction for multiple comparisons was *p* < 0.016).

The increase in the response size of P1 mediated by combined stimulation was then compared at the electrical threshold stimulating current (see arrows in Figure 5A) across the three combined stimulation optical intensities (Figure 5D). An effect of optical intensity on the response amplitude increase was seen (*p* = 0.002, one-way RM ANOVA, *n* = 6), where *post-hoc* analysis showed that there was a statistically significant difference between subthreshold and suprathreshold optical intensities (*p* = 0.01) along with parathreshold and suprathreshold optical intensities (*p* = 0.04). The change in response amplitude at each optical intensity was then tested against no increase. Combined stimulation using optical stimulation at subthreshold intensities generated no change to the neural response amplitude (*p* = 0.41). However, for combined stimulation using parathreshold and suprathreshold optical intensities, there was a statistically significant 2.4 dB ± 0.5 and 5.5 ± 1 dB increase in the hybrid ECAP response amplitude, respectively, compared to electrical-only stimulation (*p* = 0.004 and *p* = 0.003, paired t-test, adjusted statistical power after Bonferroni correction for multiple comparisons was *p* < 0.016; Figure 5D).

## Discussion

To exploit the selectivity of optogenetics and the efficiency of electrical stimulation, we explored combined stimulation in the sciatic nerve, a somatic peripheral nerve consisting of mixed fiber types. In the transgenic mouse line used in this study, the excitatory opsin ChR2-H134R was expressed in a proportion of cells in the dorsal root ganglia, classified as Aα/Aβ fibers based on conduction velocity and immunohistochemistry. When applying combined stimulation with near-threshold light, there was a significant reduction in the threshold of electrical stimulation and significant increases in the response amplitude of Aα/Aβ fibers and the therapeutic window between the neural response and myogenic activity. Since the selective increase of the activity of non-nociceptive Aβ fibers in the sciatic nerve can result in analgesia through the gate control theory mechanism, this data could have applications for chronic pain.

### ChR2-H134R expression in Aα/Aβ fibers of the sciatic nerve

The sciatic nerve is a complex, mixed somatic nerve that contains different populations of fast firing, myelinated afferents (sensory) that act as proprioceptors (Aα fibers), cutaneous mechanoreceptors that report on light touch, pressure, and vibration (Aβ fibers), as well as sharp pain (Aδ fibers), while diffuse pain is mediated by slow firing unmyelinated C-fibers. Similarly, somatic efferents (motor fibers) that control muscle are fast firing, myelinated fibers that have low electrical thresholds and require little electrical charge to be activated (Freeman et al., 2004; Snell, 2010). In this study, ChR2-H134R expression was regulated by Cre-parvalbumin. Parvalbumin, a small stable calcium binding protein, is chiefly expressed in Aα proprioceptors, but also Aβ low threshold mechanoreceptor fibers of the sciatic nerve (Arber et al., 2000; De Nooij et al., 2013; Walters et al., 2019). Our results supported this, with ChR2-H134R expression (as identified by the EYFP tag) detected in a subset of large-diameter NF200-positive DRG neurons, which is indictive of large, myelinated fibers. Although a proportion of ChR2-H134R-positive DRG neurons were medium diameter and NF200-negative, CGRP expression was not detected in these cells indicating that they are not peptidergic nociceptors (Iyengar et al., 2017). The light-responsive fibers in our ChR2-H134R mouse model had a conduction velocity of 34 m/s, which was similar to the conduction velocity of P1 evoked by electrical stimulation (28 m/s), and further supports their classification as Aα and/or Aβ fibers, which are reported to have a conduction velocity in the range of 13–35 m/s (Ruscheweyh et al., 2007). The conduction velocity border between Aβ and Aδ-fibers is 13 m/s making it unlikely that the light responding neural population are Aδ fibers.

### Responses to optical and electrical stimuli

Responses to light were detected in ChR2-H134R transgenic mice and not in the wild-type mouse indicating that the response is specific to opsin expression and not a result of heat generated from the light stimulus. The average latency of the response to the optical stimulus was longer than the electrical P1 response (1.98 ms versus 0.33 ms). This 1.65 ms difference can be attributed to several differences in the mechanisms of activation for the two stimulation modalities. Electrical stimulation directly induces a voltage change across the membrane to activate native voltage-gated ion channels allowing ions to flow down their electrochemical gradients, thus triggering an action potential (Rattay, 1999). In contrast, optogenetic stimulation must first activate the relatively slow-responding opsins to induce a depolarization current sufficient to create the voltage change that will activate the voltage-gated ion channels, and in turn trigger an action potential (Guru et al., 2015). The latency difference we observed is on the same scale as the channel opening time, which previous studies report to be 1.92 ms for ChR2-H134 when using 19.8 mW/mm^2^ optical power in HEK293 cells (Lin et al., 2009). At near-threshold levels (e.g., 6.7 mW, 1 ms optical pulse), the optical power density produced by the laser in our study was estimated to be 114 mW/mm^2^, which may account for the slightly faster channel opening kinetics observed here. Other factors, such as quality and quantity of opsin expression, are likely to have also affected the latency difference (Lin, 2011). While electrical stimulation evoked multiple neural responses and a myogenic response, optical stimulation only ever gave rise to a single detectable response.

### Combined stimulation

The use of suprathreshold light prior to the electrical stimulus had the effect of reducing the electrical threshold for activation in the optogenetically modified fibers of the sciatic nerve. A similar, but reversed, phenomenon was observed in the rat sciatic nerve, whereby electrical stimulation was combined with infrared neural stimulation to reduce optical thresholds to overcome the high-power requirements of infrared neural stimulation (Duke et al., 2009, 2012). However, while infrared neural stimulation does not require any genetic modification of the nerve, it does not allow for selective neuromodulation thus limiting the utility of this approach to selectively activate specific neural populations for therapeutic outcomes.

The difference between the P1 Aα/Aβ response threshold and P2 myogenic activity threshold for electrical stimulation was only 0.66 dB, giving a very limited range of current that can be applied before the muscles are inadvertently activated. Combined stimulation with near-threshold light intensity expanded the therapeutic stimulation window by 3.25 dB when using the 1.5 ms delay. While optical-only stimulation can be used to selectively modulate Aα/Aβ fibers, the need for long-term light delivery at high intensities could cause unacceptable local tissue heating (Stujenske et al., 2015), instigating recommendations for precautionary measures to reduce effects of heat on the nerve, such as pulsing the light, reducing the duty cycle, using larger optical fibers, and using longer wavelengths of light. Likewise, loss of the OCAP response was observed when optical powers above 15 mW were used but could be recovered at a different position on the nerve, suggesting localized desensitization of the opsin by strong light intensities (Nagel et al., 2003). Our results suggest that combining optogenetic stimulation with electrical stimulation enables the use of light at near-threshold levels while maintaining the response size and therapeutic effect, thus increasing safety. The increased therapeutic stimulation window, as measured between the Aα/Aβ fibers and myogenic activity in this study, is also expected to apply to other unwanted responses such as pain fiber activity but is yet to be validated. The improved dynamic range achieved in this study could theoretically allow the level of neural activation to be more precisely modulated to achieve the desired level of activation, as defined by the alleviation of pain, with fewer side-effects.

Conventional electrical spinal cord stimulation to treat pain is applied at stimulation rates of at least 40 Hz, sometimes much faster (De Ridder et al., 2010). Optogenetic stimulation rates are limited by opsin kinetics, with the H134R variant used in this study exhibiting a relatively slow closing time of 18 ms that limits the rate at which stimuli can be applied (Nagel et al., 2005). Long-term high rate optogenetic stimulation is less reliable compared to electrical stimulation, and the response size decreases over the duration of the pulse trains, as shown in the auditory system (Hart et al., 2020; Thompson et al., 2020; Ajay et al., 2023). However, when optogenetic stimulation is combined with electrical stimulation, the reliability of firing increases (Hart et al., 2020; Thompson et al., 2020) and can be ‘tuned’ to have response properties that are more like electrical responses or more like optical responses by adjusting the ratio of optical and electrical components of the combined stimulus (Ajay et al., 2023). Reliable control of neuromodulation would be essential to treat a condition such as pain, so keeping the optical stimuli near threshold helps to maintain the responses during repetitive stimulation.

Combined stimulation selectively increased the response amplitude in the Aα/Aβ fiber population of the sciatic nerve, even when using light at parathreshold levels. There was no effect of combined stimulation on the Aα/Aβ response size when using higher electrical stimulating levels, as the response was already near saturation. However, such high levels of electrical stimulation are rarely used in a clinical setting because they tend to be above the myogenic threshold or cause other off-target effects. In this study we consciously used stimulation levels that were predominantly below the myogenic threshold. Combined stimulation may also be useful for increasing the response amplitude when weaker cell-specific promoters are used for selective expression, or when weaker light sources such as inorganic LEDs are used, which may increase their utility and create new opportunities for clinical optogenetic stimulation strategies.

We found that the optimal delay between the optical and electrical stimuli was 1.5 ms, similar to our findings for combined stimulation in the auditory system (Thompson et al., 2020). Following the onset of the optical stimulus, photocurrents are generated in the modified nerve fibers, which can lead to facilitation, or summation, when combined with electrical stimulation. The closing kinetics of the ChR2-H134R opsin are reasonably slow (Ƭ_off_ ~ 18 ms) (Berndt et al., 2011), but our results suggest that the peak influence of the optical stimulus on action potentials occurs shortly after channel opening.

Three levels of optical intensity were used for the combined stimulus (subthreshold, parathreshold, and suprathreshold). Combined stimulation had a significant effect on response size when the light intensity was at parathreshold or suprathreshold levels, but not subthreshold intensity levels. In contrast, combined stimuli consisting of subthreshold optical and electrical stimuli was sufficient to evoke activity in the auditory system and reduced electrical thresholds for activation (Thompson et al., 2020). Nevertheless, the use of parathreshold or near-threshold optical intensities combined with low levels of electrical stimulation reduces the overall required light exposure, aiding the development of devices that can safely and effectively deliver sufficient light to the nerve.

### Potential application

The ability to selectively modulate Aα/Aβ fibers without activating unwanted fibers is critical to many applications, one of which is suppression of chronic pain. In neuropathic pain conditions, activity in normally innocuous Aα/Aβ fibers can generate pain (mechanical allodynia). However, in non-neuropathic chronic pain conditions, which includes osteoarthritis, low back pain, and irritable bowel syndrome, selective activation of touch receptors has been shown to reduce pain signaling when optogenetic stimulation was provided concurrently with activation of nociceptors (Arcourt et al., 2017). This is in line with gate control theory of pain in which it is proposed that activity in non-nociceptive fibers inhibits nociceptive transmission in the spinal cord (Melzack and Wall, 1965). Despite this, the role of Aβ fibers may be more diverse, with approximately 12% high threshold mechanoreceptors in monkey skin being nociceptors and contributing to fast pain responses (Treede et al., 1998), and a similar population was identified in humans (Nagi et al., 2019). Hence, it will be important to assess selective activation of Aβ fibers for suppression of chronic pain *via* behavioral pain assays.

### Limitations of the study

The electrical threshold for the Aα/Aβ response varied between animals, which could be caused by differences in temperature, electrode contact with the nerve, and the amount of fluid in the preparation. Nevertheless, all threshold measurements were relative to other responses in the same animal, and electrical threshold during the 5 h of recording were stable and within 50 μA. A transgenic mouse model was used for ChR2-H134R opsin expression in Aα/Aβ fibers in this study. Clinical application would require a safe method for genetic modification such as the use of adeno-associated virus (AAV) transduction *via* localized injection into the nerve (Towne et al., 2009), or an effector organ such as the muscle or skin in which selective expression in motor neurons was demonstrated (Towne et al., 2013). AAV-based transduction methods would undoubtedly lead to more variable expression of the opsin and is unlikely to be expressed in every nerve fiber of the desired neural population, unlike the transgenic mouse model used here. This may lead to reduced impact of combined stimulation, as was found when comparing transgenic and AAV-injected models in the auditory system (Richardson et al., 2021).

### Clinical considerations

Long-term optogenetic neuromodulation relies on consistent opsin expression, but loss of optogenetic responses over time may occur, due to down-regulation of opsin expression or immunogenicity to opsins causing neuronal death (Maimon et al., 2018a,b). Unlike immune-privileged sites like the retina or the cochlea, long-term opsin expression in the peripheral nervous system will be more challenging and may require immunosuppression or re-designing opsins to be less immunogenic. Secondly, optogenetic neuromodulation also relies on stable implantation of a hybrid device on a peripheral nerve, with minimal tissue reaction that may otherwise impede the delivery of light or electrical current to the nerve. There are numerous examples of nerve cuff or wireless devices incorporating electrodes and micro-LEDs that have been implanted in freely moving animals for safe, long-term stimulation of the peripheral nervous system (Payne et al., 2018; Spencer et al., 2018; Zhang et al., 2019), while other studies are exploring organic LEDs that can conform to the peripheral nerve and reduce potential mechanical damage (Kim et al., 2020).

## Conclusion

Combining selective optogenetic stimulation with electrical stimulation in the sciatic nerve reduced electrical stimulation thresholds and increased the response size exclusively in Aα/Aβ fibers. Since the myogenic response threshold did not change, the combined stimuli increased the therapeutic window between the specific Aα/Aβ neural response and the other unwanted neural responses that often accompany electrical stimulation of mixed peripheral nerves, measured here as myogenic activity. These effects of combined stimulation were observed using light intensities that were at threshold or just above threshold, thus increasing safety and reducing power requirements compared to optical-only stimulation methods while maintaining the selectivity of optogenetic stimulation. Combined stimulation has the potential to increase selectivity of neuromodulation for a range of conditions, including chronic pain, leading to safer and more efficacious stimulation with fewer off-target effects.

## Data availability statement

The raw data supporting the conclusions of this article will be made available by the authors, without undue reservation.

## Ethics statement

The animal study was reviewed and approved by St Vincent’s Hospital (Melbourne) Animal Ethics Committee. The use and care of animals in this study follow the Guidelines to Promote the Wellbeing of Animals used for Scientific Purposes (2013), the Australian Code for Care and Use of Animals for Scientific Purposes (8th edition, 2013) and the Prevention of Cruelty to Animals Amendment Act (2015).

## Author contributions

JVM, EAA, SCP, EPT, ACT, JBM, AKW, JBF, and RTR made substantial, direct, and intellectual contributions to the study and manuscript. RTR, AKW, JBF, EAA, JVM, and SCP contributed to concept and design of the research described in the manuscript. All authors contributed to acquisition of data and or analysis and interpretation of data. All authors contributed to the article and approved the submitted version.

## Funding

Research reported in this publication was supported by the Bionics Institute Incubation Fund. The Bionics Institute acknowledge the support they receive from the Victorian Government through its Operational Infrastructural Support Program.

## Conflict of interest

The authors declare that the research was conducted in the absence of any commercial or financial relationships that could be construed as a potential conflict of interest.

## Publisher’s note

All claims expressed in this article are solely those of the authors and do not necessarily represent those of their affiliated organizations, or those of the publisher, the editors and the reviewers. Any product that may be evaluated in this article, or claim that may be made by its manufacturer, is not guaranteed or endorsed by the publisher.
